# 2-[6-(4-Bromo­phen­yl)imidazo[2,1-*b*][1,3]thia­zol-3-yl]-*N*-[8-(4-hy­droxy­phen­yl)-2-methyl-3-oxo-1-thia-4-aza­spiro­[4.5]decan-4-yl]acetamide ethanol disolvate

**DOI:** 10.1107/S1600536812015371

**Published:** 2012-04-21

**Authors:** Mehmet Akkurt, Elif Gürsoy, Nuray Ulusoy Güzeldemirci, Sevim Türktekin-Çelikesir, Muhammad Nawaz Tahir

**Affiliations:** a Department of Physics, Faculty of Sciences, Erciyes University, 38039 Kayseri, Turkey; b Department of Pharmaceutical Chemistry, Faculty of Pharmacy, University of Istanbul, 34116 Beyazıt, Istanbul, Turkey; c Department of Physics, University of Sargodha, Sargodha, Pakistan

## Abstract

In the title compound, C_28_H_27_BrN_4_O_3_S_2_·2C_2_H_6_O, the cyclo­hexane ring adopts a chair conformation. The imidazo[2,1-*b*][1,3]thia­zole ring system is essentially planar with a dihedral angle of 1.1 (2)° between the thia­zole and imidazole rings. The mean plane of this ring system makes dihedral angles of 8.11 (16) and 79.43 (17)°, respectively, with the bromo- and hy­droxy-substituted benzene rings. In the 5-methyl-1,3-thia­zolidin-4-one group, the S atom, the methyl group and the ring C atoms bonded to them are disordered over two sets of sites with refined occupancies of 0.610 (19) and 0.390 (19). The crystal structure features N—H⋯O, O—H⋯O, O—H⋯N and C—H⋯O hydrogen bonds and C—H⋯π inter­actions. Furthermore, two weak π–π stacking inter­actions [centroid–centroid distances = 3.967 (3) and 3.892 (2) Å] are also observed.

## Related literature

For the biological activity of imidazo[2,1-*b*][1,3]thia­zole derivatives, see: Barradas *et al.* (2008 ▶); Juspin *et al.* (2010 ▶). For our previous papers on the synthesis of imidazo[2,1-*b*]thia­zoles, see: Gürsoy & Ulusoy Güzeldemirci (2007 ▶); Ulusoy Güzeldemirci & Küçükbasmacı (2010 ▶), and for their crystal structures, see: Akkurt *et al.* (2007 ▶, 2008 ▶, 2011 ▶). For standard bond lengths, see: Allen *et al.* (1987 ▶). For ring-puckering analysis, see: Cremer & Pople (1975 ▶).
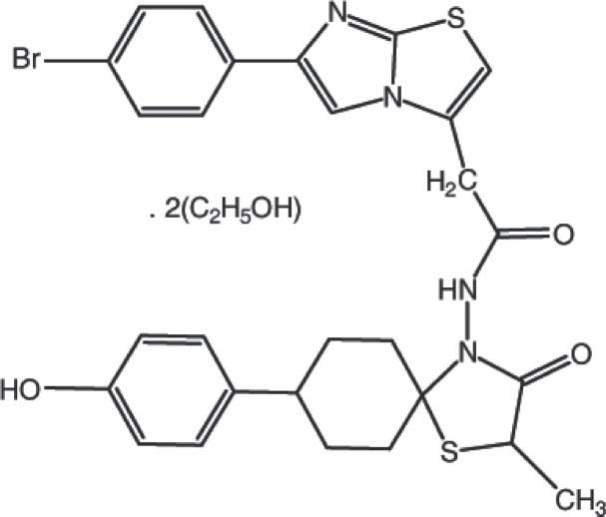


## Experimental

### 

#### Crystal data

C_28_H_27_BrN_4_O_3_S_2_·2C_2_H_6_O
*M* *_r_* = 703.71
Monoclinic, 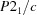

*a* = 14.9549 (15) Å
*b* = 13.2642 (11) Å
*c* = 17.9393 (17) Å
β = 109.015 (3)°
*V* = 3364.4 (5) Å^3^
*Z* = 4
Mo *K*α radiation
μ = 1.39 mm^−1^
*T* = 296 K
0.35 × 0.25 × 0.22 mm

#### Data collection

Bruker Kappa APEXII CCD diffractometer
Absorption correction: multi-scan (*SADABS*; Bruker, 2005 ▶) *T*_min_ = 0.665, *T*_max_ = 0.736
27860 measured reflections
6971 independent reflections
2926 reflections with *I* > 2σ(*I*)
*R*_int_ = 0.066

#### Refinement

*R*[*F* ^2^ > 2σ(*F* ^2^)] = 0.056
*wR*(*F* ^2^) = 0.150
*S* = 1.00
6971 reflections
428 parameters
12 restraints
H-atom parameters constrained
Δρ_max_ = 0.44 e Å^−3^
Δρ_min_ = −0.36 e Å^−3^

### 

Data collection: *APEX2* (Bruker, 2009 ▶); cell refinement: *SAINT* (Bruker, 2009 ▶); data reduction: *SAINT*; program(s) used to solve structure: *SHELXS97* (Sheldrick, 2008 ▶); program(s) used to refine structure: *SHELXL97* (Sheldrick, 2008 ▶); molecular graphics: *ORTEP-3* (Farrugia, 1997 ▶) and *PLATON* (Spek, 2009 ▶); software used to prepare material for publication: *WinGX* (Farrugia, 1999 ▶) and *PLATON*.

## Supplementary Material

Crystal structure: contains datablock(s) global, I. DOI: 10.1107/S1600536812015371/is5113sup1.cif

Structure factors: contains datablock(s) I. DOI: 10.1107/S1600536812015371/is5113Isup2.hkl

Supplementary material file. DOI: 10.1107/S1600536812015371/is5113Isup3.cml

Additional supplementary materials:  crystallographic information; 3D view; checkCIF report

## Figures and Tables

**Table 1:** Hydrogen-bond geometry (Å, °) *Cg*5 and *Cg*7 are the centroids of the C1–C6 and C23–C28 benzene rings, respectively.

*D*—H⋯*A*	*D*—H	H⋯*A*	*D*⋯*A*	*D*—H⋯*A*
N3—H3⋯O5^i^	0.86	1.92	2.771 (5)	170
O3—H3*A*⋯O2^ii^	0.82	1.91	2.713 (5)	164
O4—H4*A*⋯N1^iii^	0.82	2.07	2.857 (5)	161
O5—H5*A*⋯O4^iv^	0.82	1.84	2.655 (5)	174
C31—H31*B*⋯O1	0.96	2.49	3.312 (8)	144
C15*A*—H15*A*⋯*Cg*7^iv^	0.98	2.81	3.772 (14)	167
C24—H24⋯*Cg*5	0.93	2.69	3.600 (4)	168

Symmetry codes: (i) 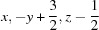; (ii) 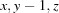; (iii) 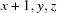; (iv) 
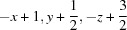
.

**Fig. 1.**
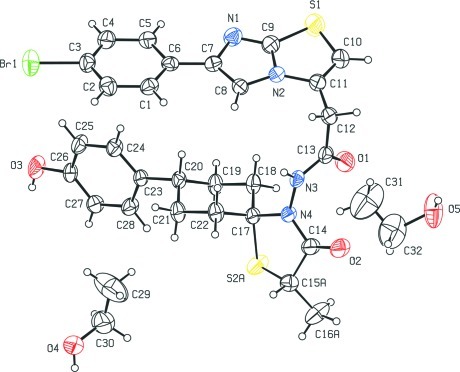
The molecular structure of the title compound. Displacement ellipsoids for non-H atoms are drawn at the 30% probability level. For clarity, only the major components of disorder are shown.

**Fig. 2.**
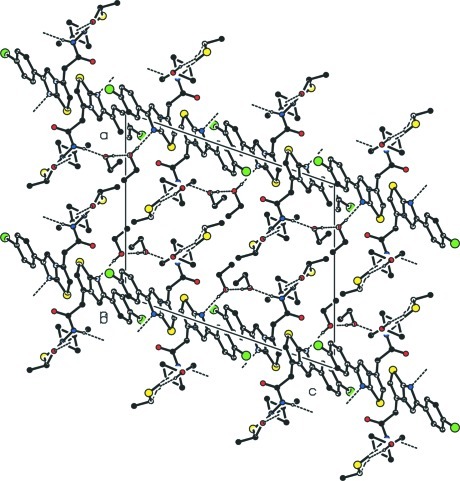
A packing diagram and hydrogen bonding of the title compound, viewed down the b axis. H atoms not involved in hydrogen bonds have been omitted for clarity. Only the major components of disorder are shown.

## supplementary crystallographic information

### Comment

Imidazo[2,1-*b*][1,3]thiazole derivatives have demonstrated a broad range of biological activities, including antiviral (Barradas *et al.*, 2008) and antibacterial (Juspin *et al.*, 2010). In connection with our previous papers on the synthesis of imidazo[2,1-*b*]thiazoles (Gürsoy & Ulusoy Güzeldemirci, 2007; Ulusoy Güzeldemirci & Küçükbasmacı, 2010) and their crystal structures (Akkurt *et al.*, 2007, 2008, 2011), we report here the crystal structure of the title spiro derivative, 2-[6-(4-bromophenyl)imidazo[2,1-*b*][1,3]thiazol-3-yl]-*N*-(8-(4-hydroxyphenyl)-2-methyl-1-thia-4-azaspiro[4.5]decan-3-one)acetamide ethanol disolvate (Fig. 1).

In the title compound (I), the S1/N2/C9–C11 thiazole and N1/N2/C7–C9 imidazole rings of the imidazo[2,1-*b*][1,3]thiazole group (S1/N1/N2/C7–C11) make a dihedral angle of 1.1 (2)° with each other. The imidazo[2,1-*b*][1,3]thiazole group makes dihedral angles of 8.11 (16) and 79.43 (17)°, with the benzene rings which have the bromo atom and the hydroxyl group, respectively. The dihedral angle between these two benzene rings is 86.7 (2)°. The bond lengths and bond angles in (I) are comparable with the values reported in related structures (Allen *et al.*, 1987; Akkurt *et al.*, 2007; 2008; 2011).

In the disordered 1,3-thiazolidine group, the S2A/N4/C14/C15A/C17 five-membered ring, with the major components of the disorder, has a twisted conformation on the S2A—C15A bond [puckering parameters (Cremer & Pople, 1975): Q(2) = 0.360 (10) Å, φ(2) = 195.4 (11) °], and the S2B/N4/C14/C15B/C17 ring, with the minor components of the disorder, adopts an envelope conformation with the C15B atom at the flap [Q(2) = 0.315 (14) Å, φ(2) = 66.3 (16) °].

In the crystal structure, molecules are connected by intermolecular N—H···O, O—H···O, O—H···N and C—H···O hydrogen bonds (Table 1, Fig. 2) and C—H···π interactions, forming a three dimensional network. Two weak π–π stacking interactions [*Cg*1···*Cg*5(-*x*, 1 - *y*, 1 - *z*) = 3.967 (3) Å and *Cg*3···*Cg*5(-*x*, 1 - *y*, 1 - *z*) = 3.892 (2) Å; where *Cg*1, *Cg*3 and *Cg*5 are the centroids of the S1/N2/C9—C11 thiazole, N1/N2/C7—C9 imidazole and C1—C6 benzene rings, respectively] are also observed.

### Experimental

A mixture of 6-(4-bromophenyl)-*N*-(4-(4-hydroxyphenyl)cyclohexylidene)imidazo[2,1-*b*]thiazole-3-acetohydrazide (0.005 mol) and 2-mercaptopropionic acid (0.01 mol) was refluxed in dry benzene (30 ml) using a Dean-Stark trap for 48 h. Excess benzene was evaporated *in vacuo*. The residue was triturated with saturated NaHCO_3_ until CO_2_ evolution ceased and then allowed to stand overnight. The solid thus obtained was filtered, washed with H_2_O and recrystallized from C_2_H_5_OH to yield colourless prisms of compound. Yield (%): 54. *M*.p. (K): 558–559. IR [ν, cm^-1^, KBr]: 3226, 3138 (O—H, N—H), 1724,1670 (C=O). Analysis calculated for C_28_H_27_N_4_O_3_S_2_. 2C_2_H_5_OH: C 54.62, H 5.59, N 7.96%. Found: C 54.16, H 5.75, N 7.35%.

### Refinement

All H atoms were placed geometrically with C—H = 0.93, 0.96, 0.97 and 0.98 Å for phenyl, methyl, methylene and methine H atoms, respectively, N—H = 0.86 Å and O—H = 0.82 Å and refined by using the riding model [*U*_iso_(H) = *xU*_eq_(C,O), *x* = 1.5 for methyl and hydroxyl H and 1.2 for all other H atoms]. In the 5-methyl-1,3-thiazolidin-4-one group of (I), the S2 atom, the methyl group (C16) and the C15 atom bound to them are disordered over two sets of sites with refined occupancies of 0.59 (2) and 0.41 (2). Three poorly fitted reflections (1 0 0), (1 6 1) and (-1 0 2) were omitted from the refinement. Distance restraints were applied for the ethanol molecules [C29—C30 and C31—C32 = 1.56 (2) Å, C29—O4 and C32—O5 = 1.35 (2) Å, C30—O4 and C31—O5 = 2.32 (2) Å] and the disordered ring [C15A C16A and C15B C16B 1.56 (2), C17—S2A, C17—S2B, C15A—S2A and C15B—S2B = 1.82 (2) Å].

### Crystal data

**Table:**

C_28_H_27_BrN_4_O_3_S_2_·2C_2_H_6_O	*F*(000) = 1464
*M**_r_* = 703.71	*D*_x_ = 1.389 Mg m^−^^3^
Monoclinic, *P*2_1_/*c*	Mo *K*α radiation, λ = 0.71073 Å
Hall symbol: -P 2ybc	Cell parameters from 327 reflections
*a* = 14.9549 (15) Å	θ = 3.5–20°
*b* = 13.2642 (11) Å	µ = 1.39 mm^−^^1^
*c* = 17.9393 (17) Å	*T* = 296 K
β = 109.015 (3)°	Prism, white
*V* = 3364.4 (5) Å^3^	0.35 × 0.25 × 0.22 mm
*Z* = 4	

### Data collection

**Table:**

Bruker Kappa APEXII CCD diffractometer	6971 independent reflections
Radiation source: fine-focus sealed tube	2926 reflections with *I* > 2σ(*I*)
Graphite monochromator	*R*_int_ = 0.066
ω scans	θ_max_ = 26.5°, θ_min_ = 2.0°
Absorption correction: multi-scan (*SADABS*; Bruker, 2005)	*h* = −18→17
*T*_min_ = 0.665, *T*_max_ = 0.736	*k* = −16→14
27860 measured reflections	*l* = −20→22

### Refinement

**Table:**

Refinement on *F*^2^	Primary atom site location: structure-invariant direct methods
Least-squares matrix: full	Secondary atom site location: difference Fourier map
*R*[*F*^2^ > 2σ(*F*^2^)] = 0.056	Hydrogen site location: inferred from neighbouring sites
*wR*(*F*^2^) = 0.150	H-atom parameters constrained
*S* = 1.00	*w* = 1/[σ^2^(*F*_o_^2^) + (0.059*P*)^2^ + 0.1004*P*] where *P* = (*F*_o_^2^ + 2*F*_c_^2^)/3
6971 reflections	(Δ/σ)_max_ < 0.001
428 parameters	Δρ_max_ = 0.44 e Å^−^^3^
12 restraints	Δρ_min_ = −0.36 e Å^−^^3^

### Special details

**Table:**

Geometry. Bond distances, angles *etc*. have been calculated using the rounded fractional coordinates. All su's are estimated from the variances of the (full) variance-covariance matrix. The cell e.s.d.'s are taken into account in the estimation of distances, angles and torsion angles
Refinement. Refinement on *F*^2^ for ALL reflections except those flagged by the user for potential systematic errors. Weighted *R*-factors *wR* and all goodnesses of fit *S* are based on *F*^2^, conventional *R*-factors *R* are based on *F*, with *F* set to zero for negative *F*^2^. The observed criterion of *F*^2^ > σ(*F*^2^) is used only for calculating -*R*-factor-obs *etc*. and is not relevant to the choice of reflections for refinement. *R*-factors based on *F*^2^ are statistically about twice as large as those based on *F*, and *R*-factors based on ALL data will be even larger.

### Fractional atomic coordinates and isotropic or equivalent isotropic displacement parameters (Å^2^)

**Table:**

	*x*	*y*	*z*	*U*_iso_*/*U*_eq_	Occ. (<1)
Br1	0.09539 (5)	0.08007 (4)	0.42261 (3)	0.0994 (3)	
S1	−0.10100 (11)	0.59938 (11)	0.71949 (9)	0.0991 (7)	
S2A	0.4804 (7)	0.5211 (7)	0.8893 (6)	0.096 (3)	0.610 (19)
O1	0.2141 (2)	0.7185 (2)	0.8423 (2)	0.0855 (16)	
O2	0.4304 (2)	0.79805 (19)	0.8429 (2)	0.0910 (13)	
O3	0.3050 (3)	−0.09257 (17)	0.72779 (19)	0.0849 (15)	
N1	−0.0424 (3)	0.4494 (3)	0.6353 (2)	0.0689 (17)	
N2	0.0378 (3)	0.5929 (2)	0.66586 (19)	0.0559 (14)	
N3	0.2814 (3)	0.6861 (2)	0.74894 (18)	0.0506 (13)	
N4	0.3622 (2)	0.6463 (2)	0.80216 (19)	0.0509 (11)	
C1	0.1150 (3)	0.3626 (3)	0.5243 (2)	0.0609 (17)	
C2	0.1293 (3)	0.2796 (3)	0.4821 (2)	0.0668 (19)	
C3	0.0752 (4)	0.1946 (3)	0.4789 (3)	0.0656 (19)	
C4	0.0080 (4)	0.1910 (3)	0.5150 (3)	0.0671 (19)	
C5	−0.0056 (3)	0.2744 (3)	0.5564 (2)	0.0610 (17)	
C6	0.0481 (3)	0.3622 (3)	0.5622 (2)	0.0506 (16)	
C7	0.0329 (3)	0.4491 (3)	0.6061 (2)	0.0537 (17)	
C8	0.0823 (3)	0.5370 (3)	0.6244 (2)	0.0525 (16)	
C9	−0.0363 (3)	0.5371 (4)	0.6704 (3)	0.0674 (19)	
C10	−0.0244 (4)	0.7006 (4)	0.7329 (3)	0.079 (2)	
C11	0.0444 (4)	0.6885 (3)	0.7021 (3)	0.0635 (17)	
C12	0.1249 (3)	0.7561 (3)	0.7072 (3)	0.0647 (16)	
C13	0.2113 (3)	0.7209 (3)	0.7744 (3)	0.058 (2)	
C14	0.4321 (3)	0.7062 (3)	0.8446 (3)	0.0741 (19)	
C15A	0.5213 (9)	0.6475 (5)	0.8785 (8)	0.059 (4)	0.610 (19)
C16A	0.5789 (17)	0.6842 (17)	0.9598 (11)	0.090 (6)	0.610 (19)
C17	0.3723 (3)	0.5360 (2)	0.8059 (2)	0.0484 (15)	
C18	0.2902 (3)	0.4860 (3)	0.8227 (2)	0.0588 (18)	
C19	0.2988 (3)	0.3717 (2)	0.8228 (2)	0.0610 (18)	
C20	0.3065 (3)	0.3332 (2)	0.7463 (2)	0.0506 (15)	
C21	0.3890 (3)	0.3819 (3)	0.7294 (2)	0.0629 (18)	
C22	0.3829 (3)	0.4969 (2)	0.7299 (2)	0.0605 (16)	
C23	0.3094 (3)	0.2193 (2)	0.7425 (2)	0.0478 (15)	
C24	0.2489 (3)	0.1687 (3)	0.6804 (2)	0.0575 (16)	
C25	0.2484 (3)	0.0647 (3)	0.6751 (3)	0.0649 (18)	
C26	0.3112 (3)	0.0097 (3)	0.7335 (2)	0.0562 (16)	
C27	0.3751 (3)	0.0577 (3)	0.7953 (3)	0.0600 (16)	
C28	0.3738 (3)	0.1618 (3)	0.7991 (2)	0.0619 (16)	
C16B	0.594 (2)	0.689 (3)	0.940 (2)	0.097 (10)	0.390 (19)
S2B	0.4827 (10)	0.5145 (10)	0.8849 (9)	0.091 (5)	0.390 (19)
C15B	0.4954 (10)	0.6441 (7)	0.9219 (13)	0.045 (5)	0.390 (19)
O4	0.7754 (2)	0.4226 (3)	0.51981 (19)	0.0853 (14)	
C29	0.6290 (5)	0.5002 (7)	0.4923 (4)	0.198 (5)	
C30	0.7073 (4)	0.4545 (5)	0.5521 (3)	0.110 (3)	
O5	0.2790 (4)	0.7871 (3)	1.0951 (2)	0.125 (2)	
C31	0.2786 (7)	0.6425 (6)	1.0282 (4)	0.206 (5)	
C32	0.3415 (7)	0.7079 (5)	1.0729 (5)	0.172 (5)	
H2	0.17430	0.28140	0.45660	0.0800*	
H3A	0.35030	−0.11790	0.76170	0.1270*	
H4	−0.02830	0.13320	0.51180	0.0810*	
H3	0.27550	0.68890	0.69970	0.0610*	
H1	0.15160	0.42000	0.52710	0.0730*	
H10	−0.03100	0.75880	0.75950	0.0950*	
H12A	0.10920	0.82480	0.71680	0.0780*	
H12B	0.13850	0.75480	0.65790	0.0780*	
H15A	0.55890	0.64780	0.84290	0.0710*	0.610 (19)
H16A	0.63560	0.64480	0.97920	0.1350*	0.610 (19)
H5	−0.05160	0.27190	0.58100	0.0730*	
H8	0.13540	0.55530	0.61140	0.0630*	
H18A	0.23160	0.50610	0.78300	0.0710*	
H18B	0.28820	0.50830	0.87360	0.0710*	
H19A	0.35430	0.35110	0.86580	0.0730*	
H19B	0.24380	0.34190	0.83150	0.0730*	
H20	0.24920	0.35490	0.70450	0.0610*	
H21A	0.39040	0.35960	0.67830	0.0750*	
H21B	0.44740	0.36050	0.76880	0.0750*	
H22A	0.43960	0.52520	0.72320	0.0720*	
H22B	0.32920	0.51880	0.68580	0.0720*	
H24	0.20640	0.20540	0.64010	0.0690*	
H25	0.20580	0.03230	0.63210	0.0780*	
H27	0.41900	0.02080	0.83460	0.0720*	
H28	0.41800	0.19420	0.84120	0.0740*	
H16B	0.54250	0.67740	0.99480	0.1350*	0.610 (19)
H16C	0.59520	0.75380	0.95690	0.1350*	0.610 (19)
H15B	0.47140	0.65210	0.96630	0.0540*	0.390 (19)
H16D	0.63680	0.65750	0.98590	0.1460*	0.390 (19)
H16E	0.59210	0.76030	0.94900	0.1460*	0.390 (19)
H16F	0.61640	0.67800	0.89580	0.1460*	0.390 (19)
H4A	0.82630	0.41630	0.55500	0.1280*	
H29A	0.58240	0.52160	0.51520	0.2960*	
H29B	0.60140	0.45200	0.45130	0.2960*	
H29C	0.65100	0.55740	0.47050	0.2960*	
H30A	0.73500	0.50290	0.59380	0.1320*	
H30B	0.68500	0.39730	0.57490	0.1320*	
H5A	0.25830	0.82680	1.05840	0.1880*	
H31A	0.31130	0.58920	1.01170	0.3090*	
H31B	0.23770	0.67670	0.98270	0.3090*	
H31C	0.24170	0.61480	1.05810	0.3090*	
H32A	0.37880	0.73820	1.04360	0.2060*	
H32B	0.38360	0.67520	1.11960	0.2060*	

### Atomic displacement parameters (Å^2^)

**Table:**

	*U*^11^	*U*^22^	*U*^33^	*U*^12^	*U*^13^	*U*^23^
Br1	0.1375 (6)	0.0619 (3)	0.1037 (5)	0.0105 (3)	0.0460 (4)	−0.0123 (3)
S1	0.0933 (12)	0.1140 (11)	0.1082 (12)	−0.0111 (9)	0.0580 (10)	−0.0321 (9)
S2A	0.098 (6)	0.044 (3)	0.092 (5)	0.014 (3)	−0.042 (4)	0.008 (3)
O1	0.085 (3)	0.112 (3)	0.056 (2)	0.018 (2)	0.018 (2)	−0.0131 (19)
O2	0.077 (2)	0.0332 (16)	0.136 (3)	−0.0046 (15)	−0.002 (2)	−0.0040 (16)
O3	0.114 (3)	0.0291 (15)	0.098 (3)	0.0003 (16)	0.016 (2)	−0.0035 (13)
N1	0.067 (3)	0.072 (3)	0.071 (3)	−0.012 (2)	0.027 (2)	−0.010 (2)
N2	0.051 (3)	0.060 (2)	0.052 (2)	0.0003 (19)	0.0102 (19)	−0.0076 (17)
N3	0.062 (3)	0.0352 (16)	0.046 (2)	0.0022 (17)	0.006 (2)	−0.0024 (15)
N4	0.054 (2)	0.0318 (16)	0.057 (2)	0.0033 (17)	0.0047 (19)	0.0034 (15)
C1	0.061 (3)	0.051 (3)	0.062 (3)	−0.004 (2)	0.008 (3)	0.004 (2)
C2	0.074 (4)	0.066 (3)	0.061 (3)	0.010 (3)	0.023 (3)	0.003 (2)
C3	0.076 (4)	0.049 (3)	0.064 (3)	0.009 (3)	0.012 (3)	−0.005 (2)
C4	0.067 (4)	0.050 (3)	0.080 (3)	−0.003 (2)	0.018 (3)	−0.002 (2)
C5	0.055 (3)	0.059 (3)	0.067 (3)	−0.001 (2)	0.017 (2)	−0.002 (2)
C6	0.043 (3)	0.049 (2)	0.054 (3)	0.002 (2)	0.008 (2)	0.0069 (19)
C7	0.051 (3)	0.058 (3)	0.049 (3)	−0.002 (2)	0.012 (2)	0.000 (2)
C8	0.048 (3)	0.051 (2)	0.059 (3)	0.003 (2)	0.018 (2)	0.000 (2)
C9	0.061 (4)	0.075 (3)	0.069 (3)	−0.005 (3)	0.025 (3)	−0.012 (3)
C10	0.071 (4)	0.092 (4)	0.072 (4)	0.010 (3)	0.020 (3)	−0.022 (3)
C11	0.060 (3)	0.058 (3)	0.061 (3)	0.012 (3)	0.004 (3)	−0.005 (2)
C12	0.062 (3)	0.044 (2)	0.076 (3)	0.011 (2)	0.006 (3)	−0.002 (2)
C13	0.064 (4)	0.041 (2)	0.063 (4)	0.003 (2)	0.011 (3)	−0.008 (2)
C14	0.056 (3)	0.040 (3)	0.107 (4)	0.001 (2)	0.000 (3)	−0.003 (2)
C15A	0.057 (7)	0.059 (5)	0.062 (7)	−0.003 (4)	0.019 (6)	0.005 (4)
C16A	0.114 (14)	0.070 (7)	0.060 (11)	−0.034 (8)	−0.006 (8)	0.012 (8)
C17	0.054 (3)	0.0316 (19)	0.056 (3)	0.0023 (19)	0.013 (2)	0.0024 (17)
C18	0.080 (4)	0.042 (2)	0.065 (3)	−0.005 (2)	0.038 (3)	−0.0074 (19)
C19	0.086 (4)	0.034 (2)	0.074 (3)	−0.004 (2)	0.041 (3)	−0.0009 (19)
C20	0.062 (3)	0.0323 (19)	0.054 (3)	0.006 (2)	0.014 (2)	0.0019 (18)
C21	0.083 (4)	0.038 (2)	0.077 (3)	0.003 (2)	0.039 (3)	0.000 (2)
C22	0.079 (3)	0.037 (2)	0.073 (3)	0.001 (2)	0.035 (3)	0.0052 (19)
C23	0.053 (3)	0.0334 (19)	0.055 (3)	0.002 (2)	0.015 (2)	0.0010 (19)
C24	0.069 (3)	0.042 (2)	0.056 (3)	0.003 (2)	0.013 (2)	0.005 (2)
C25	0.081 (4)	0.041 (2)	0.061 (3)	−0.004 (2)	0.007 (3)	−0.006 (2)
C26	0.073 (3)	0.028 (2)	0.069 (3)	0.000 (2)	0.025 (3)	−0.001 (2)
C27	0.064 (3)	0.040 (2)	0.069 (3)	0.005 (2)	0.012 (3)	0.008 (2)
C28	0.066 (3)	0.038 (2)	0.070 (3)	−0.003 (2)	0.006 (2)	0.001 (2)
C16B	0.086 (17)	0.122 (17)	0.067 (18)	0.001 (14)	0.002 (14)	0.065 (14)
S2B	0.096 (10)	0.042 (5)	0.117 (9)	−0.006 (5)	0.010 (7)	−0.001 (5)
C15B	0.037 (9)	0.039 (6)	0.061 (11)	−0.002 (5)	0.019 (7)	−0.001 (6)
O4	0.068 (2)	0.095 (2)	0.092 (3)	−0.001 (2)	0.025 (2)	−0.015 (2)
C29	0.152 (8)	0.293 (11)	0.149 (7)	0.113 (8)	0.051 (6)	0.045 (7)
C30	0.098 (5)	0.133 (5)	0.103 (5)	0.005 (4)	0.038 (4)	−0.014 (4)
O5	0.232 (5)	0.079 (2)	0.087 (3)	0.030 (3)	0.082 (3)	0.0068 (19)
C31	0.296 (13)	0.153 (7)	0.120 (7)	−0.032 (8)	0.002 (7)	0.027 (6)
C32	0.253 (11)	0.101 (6)	0.143 (7)	0.015 (6)	0.038 (7)	0.028 (5)

### Geometric parameters (Å, º)

**Table:**

Br1—C3	1.902 (5)	C23—C24	1.361 (5)
S1—C9	1.717 (5)	C23—C28	1.380 (5)
S1—C10	1.730 (6)	C24—C25	1.383 (6)
S2A—C15A	1.817 (13)	C25—C26	1.368 (6)
S2A—C17	1.821 (11)	C26—C27	1.363 (6)
S2B—C17	1.814 (16)	C27—C28	1.383 (6)
S2B—C15B	1.830 (18)	C1—H1	0.9300
O1—C13	1.206 (6)	C2—H2	0.9300
O2—C14	1.219 (5)	C4—H4	0.9300
O3—C26	1.361 (5)	C5—H5	0.9300
O3—H3A	0.8200	C8—H8	0.9300
O4—C30	1.392 (7)	C10—H10	0.9300
O4—H4A	0.8200	C12—H12B	0.9700
O5—C32	1.542 (10)	C12—H12A	0.9700
O5—H5A	0.8200	C15A—H15A	0.9800
N1—C9	1.312 (7)	C15B—H15B	0.9800
N1—C7	1.388 (6)	C16A—H16C	0.9600
N2—C11	1.414 (5)	C16A—H16B	0.9600
N2—C8	1.367 (6)	C16A—H16A	0.9600
N2—C9	1.357 (6)	C16B—H16F	0.9700
N3—N4	1.378 (5)	C16B—H16E	0.9600
N3—C13	1.354 (6)	C16B—H16D	0.9600
N4—C14	1.336 (6)	C18—H18A	0.9700
N4—C17	1.470 (4)	C18—H18B	0.9700
N3—H3	0.8600	C19—H19B	0.9700
C1—C6	1.381 (6)	C19—H19A	0.9700
C1—C2	1.392 (6)	C20—H20	0.9800
C2—C3	1.378 (6)	C21—H21B	0.9700
C3—C4	1.362 (9)	C21—H21A	0.9700
C4—C5	1.384 (6)	C22—H22A	0.9700
C5—C6	1.399 (6)	C22—H22B	0.9700
C6—C7	1.455 (6)	C24—H24	0.9300
C7—C8	1.362 (6)	C25—H25	0.9300
C10—C11	1.327 (9)	C27—H27	0.9300
C11—C12	1.480 (7)	C28—H28	0.9300
C12—C13	1.525 (7)	C29—C30	1.439 (10)
C14—C15B	1.63 (2)	C29—H29B	0.9600
C14—C15A	1.492 (13)	C29—H29A	0.9600
C15A—C16A	1.51 (2)	C29—H29C	0.9600
C15B—C16B	1.52 (4)	C30—H30A	0.9700
C17—C18	1.510 (6)	C30—H30B	0.9700
C17—C22	1.515 (5)	C31—C32	1.338 (12)
C18—C19	1.522 (5)	C31—H31A	0.9600
C19—C20	1.504 (5)	C31—H31B	0.9600
C20—C23	1.514 (4)	C31—H31C	0.9600
C20—C21	1.508 (6)	C32—H32A	0.9700
C21—C22	1.528 (5)	C32—H32B	0.9700
			
C9—S1—C10	89.2 (3)	C4—C5—H5	119.00
C15A—S2A—C17	93.0 (6)	C6—C5—H5	119.00
C15B—S2B—C17	95.5 (9)	C7—C8—H8	127.00
C26—O3—H3A	109.00	N2—C8—H8	127.00
C30—O4—H4A	109.00	C11—C10—H10	123.00
C32—O5—H5A	109.00	S1—C10—H10	123.00
C7—N1—C9	104.2 (4)	C11—C12—H12A	110.00
C9—N2—C11	113.4 (4)	C11—C12—H12B	110.00
C8—N2—C11	139.8 (4)	C13—C12—H12B	110.00
C8—N2—C9	106.8 (3)	H12A—C12—H12B	108.00
N4—N3—C13	119.8 (3)	C13—C12—H12A	110.00
N3—N4—C17	117.8 (3)	C16A—C15A—H15A	111.00
C14—N4—C17	121.1 (3)	S2A—C15A—H15A	111.00
N3—N4—C14	120.9 (3)	C14—C15A—H15A	111.00
C13—N3—H3	120.00	C16B—C15B—H15B	112.00
N4—N3—H3	120.00	C14—C15B—H15B	112.00
C2—C1—C6	122.0 (4)	S2B—C15B—H15B	112.00
C1—C2—C3	118.5 (4)	C15A—C16A—H16C	109.00
C2—C3—C4	121.5 (4)	H16A—C16A—H16B	110.00
Br1—C3—C4	119.6 (3)	H16A—C16A—H16C	109.00
Br1—C3—C2	118.9 (4)	H16B—C16A—H16C	109.00
C3—C4—C5	119.2 (4)	C15A—C16A—H16B	110.00
C4—C5—C6	121.6 (4)	C15A—C16A—H16A	109.00
C5—C6—C7	120.8 (4)	C15B—C16B—H16E	110.00
C1—C6—C7	122.0 (4)	H16D—C16B—H16E	109.00
C1—C6—C5	117.2 (4)	H16D—C16B—H16F	109.00
N1—C7—C8	110.5 (4)	C15B—C16B—H16F	110.00
C6—C7—C8	129.8 (4)	H16E—C16B—H16F	109.00
N1—C7—C6	119.7 (4)	C15B—C16B—H16D	110.00
N2—C8—C7	105.9 (4)	C19—C18—H18A	109.00
N1—C9—N2	112.7 (4)	C19—C18—H18B	109.00
S1—C9—N2	112.1 (4)	H18A—C18—H18B	108.00
S1—C9—N1	135.2 (4)	C17—C18—H18B	109.00
S1—C10—C11	114.6 (4)	C17—C18—H18A	109.00
N2—C11—C10	110.7 (5)	C18—C19—H19B	109.00
N2—C11—C12	120.4 (5)	C18—C19—H19A	109.00
C10—C11—C12	128.8 (4)	C20—C19—H19B	109.00
C11—C12—C13	109.0 (4)	C20—C19—H19A	109.00
O1—C13—C12	123.3 (4)	H19A—C19—H19B	108.00
N3—C13—C12	112.8 (4)	C21—C20—H20	107.00
O1—C13—N3	123.8 (4)	C23—C20—H20	107.00
O2—C14—C15B	122.0 (6)	C19—C20—H20	107.00
N4—C14—C15B	108.4 (5)	C20—C21—H21B	109.00
O2—C14—C15A	122.7 (5)	C22—C21—H21A	109.00
N4—C14—C15A	110.6 (5)	C20—C21—H21A	109.00
O2—C14—N4	125.0 (4)	H21A—C21—H21B	108.00
C14—C15A—C16A	112.2 (11)	C22—C21—H21B	109.00
S2A—C15A—C16A	107.5 (11)	C17—C22—H22B	109.00
S2A—C15A—C14	103.8 (8)	H22A—C22—H22B	108.00
S2B—C15B—C16B	115.0 (17)	C21—C22—H22A	109.00
S2B—C15B—C14	101.8 (12)	C21—C22—H22B	109.00
C14—C15B—C16B	103.8 (16)	C17—C22—H22A	109.00
N4—C17—C18	111.6 (3)	C23—C24—H24	119.00
S2A—C17—N4	101.3 (4)	C25—C24—H24	119.00
S2A—C17—C22	112.2 (4)	C24—C25—H25	120.00
S2B—C17—N4	104.2 (5)	C26—C25—H25	120.00
S2A—C17—C18	110.5 (4)	C28—C27—H27	120.00
S2B—C17—C18	111.6 (5)	C26—C27—H27	120.00
S2B—C17—C22	108.3 (6)	C23—C28—H28	119.00
N4—C17—C22	109.8 (3)	C27—C28—H28	119.00
C18—C17—C22	111.1 (3)	O4—C30—C29	110.2 (5)
C17—C18—C19	111.4 (3)	C30—C29—H29A	109.00
C18—C19—C20	111.7 (3)	C30—C29—H29B	109.00
C21—C20—C23	112.4 (4)	H29A—C29—H29B	110.00
C19—C20—C23	113.1 (3)	H29A—C29—H29C	109.00
C19—C20—C21	110.4 (3)	C30—C29—H29C	109.00
C20—C21—C22	111.8 (3)	H29B—C29—H29C	109.00
C17—C22—C21	111.8 (3)	O4—C30—H30B	110.00
C24—C23—C28	116.6 (3)	C29—C30—H30A	110.00
C20—C23—C24	120.5 (3)	O4—C30—H30A	110.00
C20—C23—C28	122.8 (3)	H30A—C30—H30B	108.00
C23—C24—C25	122.4 (4)	C29—C30—H30B	110.00
C24—C25—C26	119.5 (4)	O5—C32—C31	103.3 (8)
C25—C26—C27	119.9 (4)	C32—C31—H31A	109.00
O3—C26—C27	122.6 (4)	C32—C31—H31B	109.00
O3—C26—C25	117.5 (4)	C32—C31—H31C	109.00
C26—C27—C28	119.3 (4)	H31A—C31—H31B	109.00
C23—C28—C27	122.2 (4)	H31A—C31—H31C	110.00
C2—C1—H1	119.00	H31B—C31—H31C	109.00
C6—C1—H1	119.00	O5—C32—H32A	111.00
C3—C2—H2	121.00	O5—C32—H32B	111.00
C1—C2—H2	121.00	C31—C32—H32A	111.00
C5—C4—H4	120.00	C31—C32—H32B	111.00
C3—C4—H4	120.00	H32A—C32—H32B	109.00
			
C9—S1—C10—C11	0.4 (4)	C4—C5—C6—C1	0.5 (6)
C10—S1—C9—N2	−0.2 (4)	C4—C5—C6—C7	−179.9 (4)
C10—S1—C9—N1	−178.8 (6)	C5—C6—C7—N1	−7.1 (5)
C15A—S2A—C17—C22	93.9 (6)	C5—C6—C7—C8	173.6 (4)
C15A—S2A—C17—C18	−141.6 (5)	C1—C6—C7—C8	−6.8 (6)
C17—S2A—C15A—C14	30.0 (8)	C1—C6—C7—N1	172.5 (4)
C17—S2A—C15A—C16A	149.0 (12)	C6—C7—C8—N2	179.6 (4)
C15A—S2A—C17—N4	−23.2 (6)	N1—C7—C8—N2	0.3 (4)
C7—N1—C9—S1	178.5 (4)	S1—C10—C11—N2	−0.6 (6)
C9—N1—C7—C6	−179.5 (4)	S1—C10—C11—C12	−175.5 (4)
C7—N1—C9—N2	−0.1 (5)	C10—C11—C12—C13	95.9 (6)
C9—N1—C7—C8	−0.1 (5)	N2—C11—C12—C13	−78.6 (5)
C8—N2—C11—C12	−6.3 (8)	C11—C12—C13—N3	113.7 (4)
C11—N2—C9—S1	−0.1 (5)	C11—C12—C13—O1	−61.9 (5)
C11—N2—C9—N1	178.9 (4)	O2—C14—C15A—C16A	50.0 (14)
C11—N2—C8—C7	−178.3 (5)	N4—C14—C15A—S2A	−28.4 (9)
C8—N2—C9—N1	0.3 (5)	N4—C14—C15A—C16A	−144.1 (11)
C9—N2—C11—C10	0.4 (6)	O2—C14—C15A—S2A	165.7 (6)
C8—N2—C9—S1	−178.7 (3)	S2A—C17—C18—C19	−70.7 (4)
C9—N2—C11—C12	175.8 (4)	N4—C17—C18—C19	177.4 (3)
C9—N2—C8—C7	−0.4 (4)	C22—C17—C18—C19	54.5 (4)
C8—N2—C11—C10	178.3 (5)	S2A—C17—C22—C21	70.8 (5)
N4—N3—C13—C12	−176.4 (3)	N4—C17—C22—C21	−177.3 (3)
C13—N3—N4—C14	−82.1 (5)	C18—C17—C22—C21	−53.4 (4)
C13—N3—N4—C17	102.9 (4)	C17—C18—C19—C20	−56.6 (4)
N4—N3—C13—O1	−0.9 (6)	C18—C19—C20—C21	56.5 (4)
C17—N4—C14—O2	177.6 (4)	C18—C19—C20—C23	−176.6 (4)
N3—N4—C14—O2	2.7 (7)	C19—C20—C21—C22	−55.2 (4)
C14—N4—C17—C22	−108.1 (4)	C23—C20—C21—C22	177.5 (3)
N3—N4—C14—C15A	−162.8 (6)	C19—C20—C23—C24	127.9 (4)
C17—N4—C14—C15A	12.1 (8)	C19—C20—C23—C28	−54.1 (6)
N3—N4—C17—S2A	−174.3 (4)	C21—C20—C23—C24	−106.2 (5)
C14—N4—C17—S2A	10.7 (6)	C21—C20—C23—C28	71.8 (5)
N3—N4—C17—C18	−56.7 (4)	C20—C21—C22—C17	54.2 (4)
C14—N4—C17—C18	128.3 (4)	C20—C23—C24—C25	−179.2 (4)
N3—N4—C17—C22	67.0 (5)	C28—C23—C24—C25	2.7 (7)
C2—C1—C6—C5	−0.2 (6)	C20—C23—C28—C27	179.4 (4)
C6—C1—C2—C3	−0.5 (6)	C24—C23—C28—C27	−2.5 (7)
C2—C1—C6—C7	−179.8 (4)	C23—C24—C25—C26	−0.7 (7)
C1—C2—C3—Br1	−179.1 (3)	C24—C25—C26—O3	176.8 (4)
C1—C2—C3—C4	0.9 (7)	C24—C25—C26—C27	−1.6 (7)
C2—C3—C4—C5	−0.6 (8)	O3—C26—C27—C28	−176.6 (4)
Br1—C3—C4—C5	179.4 (4)	C25—C26—C27—C28	1.7 (7)
C3—C4—C5—C6	−0.1 (7)	C26—C27—C28—C23	0.4 (7)

### Hydrogen-bond geometry (Å, º)

Cg5 and Cg7 are the centroids of the C1–C6 and C23–C28 benzene rings, respectively.

**Table:**

*D*—H···*A*	*D*—H	H···*A*	*D*···*A*	*D*—H···*A*
N3—H3···O5^i^	0.86	1.92	2.771 (5)	170
O3—H3*A*···O2^ii^	0.82	1.91	2.713 (5)	164
O4—H4*A*···N1^iii^	0.82	2.07	2.857 (5)	161
O5—H5*A*···O4^iv^	0.82	1.84	2.655 (5)	174
C5—H5···N1	0.93	2.53	2.864 (6)	101
C31—H31*B*···O1	0.96	2.49	3.312 (8)	144
C15*A*—H15*A*···*Cg*7^iv^	0.98	2.81	3.772 (14)	167
C24—H24···*Cg*5	0.93	2.69	3.600 (4)	168

Symmetry codes: (i) *x*, −*y*+3/2, *z*−1/2; (ii) *x*, *y*−1, *z*; (iii) *x*+1, *y*, *z*; (iv) −*x*+1, *y*+1/2, −*z*+3/2.
